# Dysregulation of the Immune System in a Natural History Study of 1299 Individuals with Down Syndrome

**DOI:** 10.21203/rs.3.rs-3647800/v1

**Published:** 2023-12-04

**Authors:** William Gansa, Kartikeya Menon, Christos Sazeides, O’Jay Stewart, Dusan BOGUNOVIC

**Affiliations:** Icahn School of Medicine at Mount Sinai

**Keywords:** Down syndrome, autoimmune, natural history, viral disease, dysregulation, inflammation

## Abstract

Dysregulation of the immune system in individuals with Down syndrome is thought to play a major role in the pathophysiology of many clinical presentations. This natural history of disease study took a comprehensive evaluation of the prevalence of different immune related diagnoses in a cohort of 1299 patients with Down syndrome compared to a 2605 control cohort of patients without Down syndrome at Mount Sinai Health System in NY, NY over the past 18 years. We conducted a stepwise analysis of the odds of receiving a diagnosis at the Chapter, Sub-chapter and Diagnosis level of the ICD-CM-10 code system. Individuals in our Down syndrome cohort had higher odds of a diagnosis with inflammatory and autoimmune presentations such as Alopecia areata (OR 6.06, p = 0.01), Other sepsis (OR 4.79, p < 0.001, Purpura and Other hemorrhagic conditions (OR 2.31, p < 0.001), and Rosacea (OR 3.11, p < 0.001). They also presented with lower odds of a diagnosis of Herpesviral infection (OR 0.42, p = 0.01), and Viral warts (OR 0.51, p = 0.04). We posit that dysregulation of the immune system in individuals with Down syndrome has impact on infectious diseases, including lowering the incidence of viral disease, and increasing its severity. Our data also suggests inflammation and autoimmune mediated diseases, in particular of the skin, is exacerbated in individuals with Down syndrome. Finally, there may be a need for greater clinical attention to non-emergent conditions within the Down syndrome patient population as those can also greatly affect quality of life.

## Introduction

Down syndrome, or Trisomy 21, is a chromosomal abnormality due to the complete or partial presence of a third copy of chromosome 21 [1]. In the United States, it occurs at a rate of roughly 1 in 700–800 live births, with more than 200,000 individuals currently living with Down Syndrome [2–3]. Prevalence has increased in the 21st century, largely due to the increased life span of individuals with Down syndrome: in 2010, a mean of 53 years and a median of 58 years as compared to 26 years and 4 years, respectively, in 1950 [4]. Much of this trend has been driven by advances in medical and surgical interventions for neonatal and pediatric individuals with Down syndrome. As recently as 1973, epidemiological studies in Sweden and the UK suggested that less than half of individuals born with Down Syndrome were expected to survive the neonatal period; in 2010, the survival rate had increased to 95% [1, 5].

Down syndrome manifests with significant phenotypic variation, although the reasons behind the heterogeneic presentation are elusive. One common hypothesis is that genetic variants in 200-protein coding genes on the extra copy of chromosome 21 may have different cis and trans effects [2, 4].

In recent years, this multi-organ multi-system syndrome has sparked a burgeoning literature describing associated comorbidities. Among individuals with Down syndrome, roughly 50% present with congenital heart defects (commonly atrial and ventricular septal defects), more than half present with obstructive sleep apnea, more than half of neonates with Down syndrome present with abnormal thyroid tests, and about 8% of pediatric individuals with Down Syndrome present with epilepsy. Additionally, individuals present with both conductive and sensorineural hearing loss, with ophthalmoplegias (commonly congenital and developmental cataracts and refractive errors), with atlantoaxial instability, with mental health disorders (such as anxiety and depressive disorders), with intellectual disability, and with early-onset Alzheimer’s disease [2, 4, 6].

The treatment of patients with Down syndrome varies by phenotype and organ system: for example, identification of congenital heart defects and gastrointestinal abnormalities should be assessed in the perinatal period, while neurodegenerative disorders and hearing deficits may be screened for throughout the lifetime [2]. To guide clinicians, a robust series of international practice guidelines exist for pediatric patients with Down Syndrome for these particular symptoms and signs, in part due to the high historic mortality in the neonatal period, and historically shorter lifespan [6].

More recently, as lifespan has dramatically increased, and early onset diseases have been better managed, it has been recognized that individuals with Down syndrome present with disorders of immune dysregulation [1, 7, 8]. Interestingly they present with both susceptibility to severe infectious diseases (despite having lower incidence of these infections), and concomitant increased levels of autoimmunity and autoinflammation, presenting as alopecia areata, type I diabetes, eczema, atopic diseases of the lung and skin as well as generalized inflammation [8, 9, 10, 11, 12].

Unlike for pediatric patients, no definitive clinical guidelines currently exist for adults with Down syndrome, especially in the context of immune dysregulation, although some efforts are in progress [2]. This may in part contribute to high mortality rates for adult individuals (> 35 years old) with Down syndrome as compared to their peers without Down syndrome. Additionally, many individuals with Down syndrome are at higher risk of chronic disease as they age: nearly 40% of individuals with Down syndrome will develop Alzheimer’s disease by the age of 55 [1].

Although some data exists describing the heterogeneous phenotypes and manifestations of Down Syndrome, less is known about how patients with Down syndrome present within the healthcare system across disparate age groups and by organ system or disease grouping. This lack of knowledge presents a roadblock in the development of substantial and effective preventative health guidelines to screen for health issues before they worsen.

Given these gaps in knowledge, we sought to develop a more comprehensive understanding of which disease manifestations are more common in individuals with Down syndrome at a large, urban tertiary/quaternary care center as compared to individuals without Down syndrome. Thus we interrogated the Electronic Health Record to ascertain which diagnoses are more likely in a Down syndrome cohort consisting of 1299 individuals over the last 18 years, as compared to an age, race, sex, and insurance-matched control cohort. Our data coincides with the sharp rise in prevalence and life expectancy of patients with Down syndrome and may shed light on how this epidemiological trend manifests in the clinic. Our findings also add to the growing understanding of the phenotype of the adult patient with Down syndrome and may contribute to the development of a robust and evidence based series of screening guidelines.

## Methods

### Data

We used 18 years of encounter data in the form of International Classification of Diseases-10-Clinical Modification (ICD-10-CM) codes from the electronic medical record of a large northeastern hospital network including eight hospital campuses and over 410 ambulatory practice locations with over 3,000,000 outpatient encounters and over 130,000 inpatient admissions. Aggregation of the data was performed by the Mount Sinai Data Warehouse using ATLAS, an open-source application developed by the Observational Health Data Sciences and Informatics team [13]. Individuals who were diagnosed in any encounter with an ICD-10-CM code were considered positive for that condition in all subsequent analyses. The Institutional Review Board of the Icahn School of Medicine at Mount Sinai approved IRB-18–00638/STUDY-18–00627.

### Cohort

The study cohort included individuals with a diagnosis of Down syndrome who were seen within the Mount Sinai Health System from 2005 to 2023. Down syndrome was defined as a ICD-10-CM code of Q90.0 “Down Syndrome”, Q90.0 “Trisomy 21, nonmosaicism, (meiotic nondisjunction),” Q90.1 “Trisomy 21 mosaicism (mitotic nondisjunction),” Q90.2 “Trisomy 21, translocation,” or Q90.9 “Down Syndrome, unspecified” [14]. Individuals with < 2 encounters with the health system were excluded from the study. We identified 1299 individuals who met the inclusion criteria for the study.

### Controls

We matched each individual in our cohort to at least one and a maximum of three age, race, sex, and insurance-matched individuals without Down syndrome. Individuals with < 2 encounters with the health system were excluded from the study. We identified 2605 individuals who met the inclusion criteria for controls.

### Analysis

We first analyzed the demographic makeup of our case versus control cohorts by age, sex, race, and insurance status (Table 1). In the cases where the insurance status or race was unknown, we removed the data from the analysis. We calculated p-values and chi squared test statistics for this portion of the analysis.

**Figure 1:**
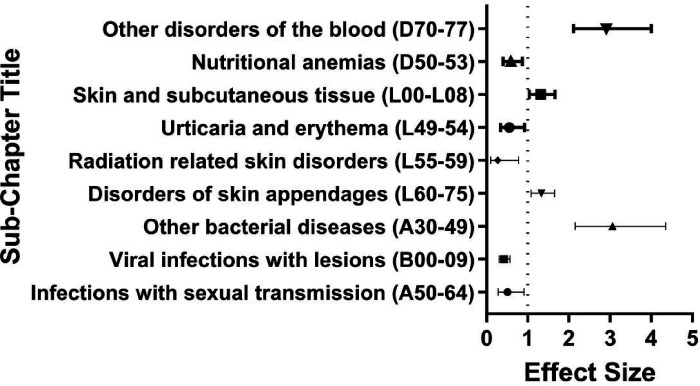
Odds Ratio by Sub-chapter Diagnosis

**Figure 2:**
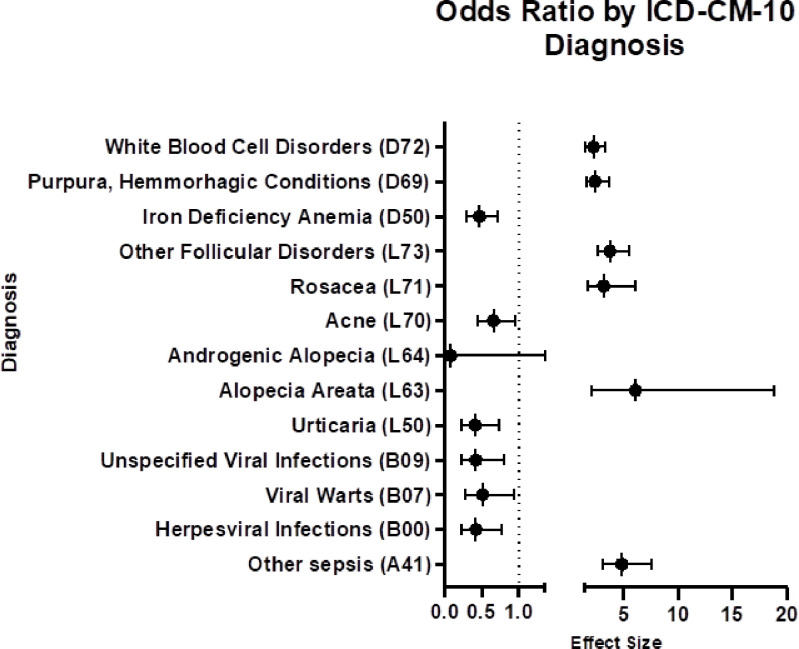
Odds Ratio by Diagnosis

**Figure 3:**
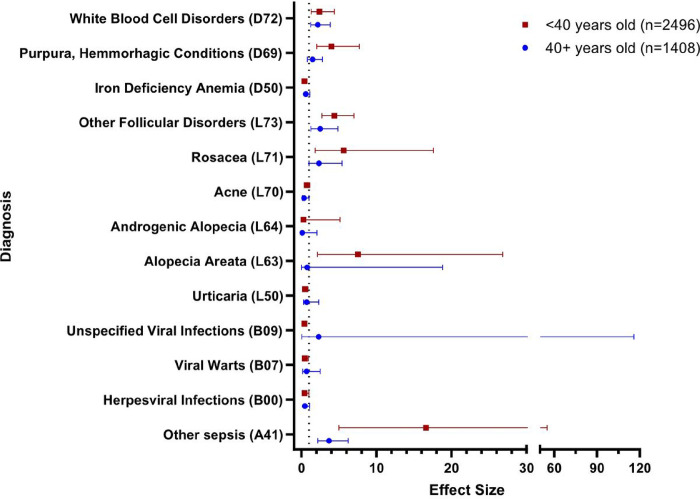
Odds of Receiving a Diagnosis Within ICD-10-CM Chapter Compared with Control in Individuals <40 years old and 40+ years old

We next analyzed our diagnostic data using the ICD-10 codes in a stepwise manner. We first analyzed the case versus control data at the ICD-10-CM chapter level (Table 2). In this analysis, any diagnosis within a predetermined range of ICD-10-CM codes was considered a positive hit. Next, we analyzed the data in selected subchapter levels, based on our findings in the previous step. In this analysis, any diagnosis within a narrower range of ICD-10-CM codes was considered a hit. We selected the three subchapters (“Certain infectious and parasitic diseases”, “Diseases of the blood and blood forming organs and certain disorders of the immune process,” and “Diseases of the skin and subcutaneous tissue”) to investigate further based on relative underreporting of these diagnoses in the literature. We chose not to pursue certain other subchapters because those disease processes are well documented in the literature [15]. We next performed an analysis at the diagnosis level based on our previous findings. In this analysis, a positive hit was determined only when the exact ICD-10-CM code was present in the patient’s electronic medical record. Finally, we split our Down syndrome and control cohort into two age bins: >40 years old and 40 + years old to analyze odds of receiving our diagnoses of interest across age groups of the population. We calculated odds ratios, p-values and chi squared test statistics for this portion of the analysis (Fig. 1, 2, 3).

## Results

### Demographics

Our case population of 1299 total individuals with Down syndrome was majority female (53%), and non-Hispanic white (51%) (Table 1). A plurality of the case population was > 40 years old (33%), with roughly equal representation from the 6–10, 11–20, 21–30, and 31–40 age groups (10–15%, respectively). The case population insurance status was evenly split between private insurance (35%) and Medicaid (35%) with a significant portion of patients on Medicare (30%).

Our control population of 2605 total individuals without Down syndrome was also majority female (57%) and non-Hispanic White (52%). A plurality of the control population was also > 40 years old (38%) and also with roughly equal representation from the 6–10, 11–20, 21–30, and 31–40 age groups (11–13%, respectively). The majority of the control population insurance status was private insurance or Medicaid (40% and 35%, respectively) with individuals insured by Medicare representing 24% of the population.

The case and control populations were not significantly different by sex, age, or race. The cohorts were significantly different by insurance status (p = 0.002, with Pearson’s chi squared test = 18.29).

### ICD-10-CM Chapter Level

At the ICD-10CM Chapter level we found that individuals in our Down syndrome cohort had lower odds of a diagnosis of “Certain infectious and parasitic diseases” (A00-B99) as compared to control individuals without Down syndrome (Odds Ratio [OR] 0.87, p = 0.06). Individuals in our Down syndrome cohort also had lower odds of a diagnosis of “Neoplasms” (C00-D49) (OR 0.36, p = < 0.001), “Diseases of the skin and subcutaneous tissue” (L00-L99) (OR 0.81, p = 0.01), “Diseases of the musculoskeletal system and connective tissue (M00-M99) (OR 0.68, p < 0.001), “Diseases of the genitourinary system” (N00-N99) (OR 0.73, p < 0.001), and “Factors influencing health status and contact with health services” (Z00-Z99) (OR 0.64, p < 0.001) (Table 2). These findings are largely in line with previous studies. Although individuals with Down syndrome are known to be at increased risk of certain neoplasms, this data suggests that overall risk may be decreased. [4, 5].

We found that individuals in our Down syndrome cohort as compared to control individuals without Down syndrome had higher odds of a diagnosis of “Diseases of the blood and blood forming organs and certain disorders of the immune process” (D50-D89) (OR 1.19, p = 0.08), “Endocrine, nutritional, and metabolic diseases” (E00-E90) (OR 1.89, p = 0.08), “Mental and behavioral disorders” (F00-F99) (OR 1.47, p < 0.001), “Diseases of the nervous system (G00-G99) (OR 1.97, p < 0.001), “Diseases of the eye and adnexa” (H00-H59) (OR 1.52, p < 0.001), “Diseases of the ear and mastoid process” (H60-H65) (OR 1.68, p < 0.001), “Diseases of the circulatory system” (I00-I99) (OR 2.02, p < 0.001), “Symptoms, signs and abnormal clinical and laboratory findings, not elsewhere classified” (R00-R99) (OR 1.29, p < 0.001), and “Injury, poisoning, and certain other consequences of external causes” (S00-T98) (OR 9.28, p < 0.001) (Table 2). Many of these findings are in line with previous studies [4, 5]. Notably, our finding of increased odds of injury and/or poisoning from external causes emphasizes the outsize role caregivers may have in the health of individuals with Down syndrome [4].

### ICD-10-CM Sub-Chapter Level

Within the sub-chapter of “Certain infectious and parasitic diseases” (A00-B99), we found that individuals in our Down syndrome cohort as compared to control individuals without Down syndrome had lower odds of a diagnosis of “Infections with a predominantly sexual mode of transmission” (A50–64) (OR 0.51, p = 0.03). While hard to prove it is reasonable to assume that behavioral aspects of individuals with Down syndrome significantly contribute to these outcomes. We also document that “Viral infections characterized by skin and mucus membrane lesions” occur at lower rates (B00-B09) (OR 0.42, p < 0.001). However, they also had higher odds of a diagnosis of “Other bacterial diseases” (A30–49) (OR 3.06, p < 0.001) (Fig. 1)

Within the sub-chapter “Diseases of the skin and subcutaneous tissue” (L00-L99), we found that individuals in our Down syndrome cohort as compared to control individuals without Down syndrome had lower odds of a diagnosis of “Radiation related disorders of the skin and subcutaneous tissue (L55-L59) (OR 0.27, p = 0.03) and “Urticaria and erythema” (L49–54) (OR 0.56, p = 0.02). However individuals with Down Syndrome had higher odds of a diagnosis of “Infections of the skin and subcutaneous tissue” (L00-L08) (OR 1.32, P = 0.03) and higher odds of a diagnosis of “Disorders of skin appendages” (L60–75) (OR 1.33, P = 0.01) (Fig. 1). To which extent the diagnoses reported as rarer are truly so in individuals with Down Syndrome is hard to ascertain as it is possible they are simply underreported by individuals themselves and underdiagnosed by the providers. In this sub-chapter, certain diagnoses may be driven by environmental versus immune factors and require further granular investigation to untangle.

Within the sub-chapter “Diseases of the blood and blood forming organs and certain disorders of the immune process” (D50-D89), we found that individuals in our Down syndrome control as compared to control individuals without Down Syndrome had lower odds of a diagnosis of “Nutritional anemias” (D50-D53) (OR 0.59, p = 0.01). However they had higher odds of a diagnosis of “Other disorders of blood and blood forming organs” (D70–77) (OR 2.91, p < 0.001) (Fig. 1). Here again, the interplay between caretakers of individuals with Down syndrome, the Down syndrome phenotype, and the medical system may be protective in certain instances such as dietary intake.

### ICD-10-CM Diagnosis Level

#### Bacterial and Viral Infections

We found that individuals in our Down syndrome cohort as compared to control individuals without Down syndrome had lower odds of a diagnosis “Herpesviral infections” (B00) (OR 0.42, p = 0.01), “Viral warts” (B07) (OR 0.51, p = 0.04), and “Unspecified viral infections characterized by skin and mucous membrane lesions” (OR 0.42, p = 0.01) (Fig. 2). The extent to which this is mediated by additional copies of type 1 interferon receptor (IFNAR) encoded by chromosome 21 contributing to a hyperactive response to key antiviral cytokine type I interferon is worth exploring further. (36813963) (32572726) (27472900) Interestingly individuals with Down syndrome had higher odds of a diagnosis of “Other sepsis” (A41), (OR 4.79, p < 0.001) (Fig. 2), which is perhaps founded in their already increased steady state of inflammation as documented by several studies [8, 16, 17, 18].

#### Blood and Blood Forming Organs and Diseases of the Immune Mechanism

We found that individuals in our Down syndrome cohort as compared to control individuals without Down syndrome had lower odds of a diagnosis of “Iron deficiency anemia” (D50) (OR 0.47, p < 0.001). However, they had higher odds of a diagnosis of “Other nutritional anemias” (D53) (OR 6.04, 0.03), “Purpura and other hemorrhagic conditions” (D69) (OR 2.31,, p < 0.001), “Other disorders of white blood cells” (D72) (OR 2.18, p = 0.000), and “Other and unspecified diseases of blood and blood forming organs” (D75) (OR 6.50,, p < 0.001) (Fig. 2). Previous studies on hematologic abnormalities in individuals with Down syndrome have largely focused on malignancies and the creation of suggested screening guidelines [2, 5]. This finding sheds light on the consequences of immune dysregulation with the hematologic system of individuals with Down syndrome, which has been less well studied.

#### Skin and Subcutaneous Tissue

We found that individuals in our Down syndrome cohort as compared to control individuals without Down syndrome had lower odds of a diagnosis of “Urticaria” (L50) (OR 0.41, p = 0.03), “Androgenic Alopecia” (L64) (OR 0.08, p = 0.08), and “Acne” (L70) (OR 0.66, p = 0.04). However, they had higher odds of a diagnosis of “ Alopecia Areata” (L63) (OR 6.06, p = 0.01), “Rosacea” (L71) (OR 3.11, p < 0.001), and “Other follicular disorders” (L73) (OR 3.70, p < 0.001) (Fig. 2). One possible explanation for these skin related disorders can be founded in the increased levels of steady state inflammation which leads to hyperactive immune system and ultimately autoimmunity, which can be both T and B cell governed [8, 11, 12]. Only detailed molecular understanding of the rogue immune system can help us first understand pathophysiology of particular diseases, and then allow us to deploy appropriate therapeutic modalities.

#### Age Binned Analysis

There were 2496 individuals in our < 40 year old cohort (case n = 872, control n = 1624) and 1408 individuals in our 40 + year old cohort (case n = 427 control n = 981). We found that the odds of a diagnosis of “Other sepsis” (A41) in our Down syndrome cohort as compared to control individuals was increased in the < 40 year old age bin (OR 16.6, p < 0.001) as compared to the 40 + year old age bin (OR 3.67, p < 0.001). Down syndrome individuals in the < 40 year old age bin also had higher odds of receiving a diagnosis of “Rosacea” (L71) [< 40 year old age bin (OR 5.65, p = 0.01) versus the 40 + year old age bin (OR 2.33, 0.05)], “Other follicular disorders” (L73) [< 40 year old age bin (OR 4.38, p < 0.001) versus the 40 + year old age bin (OR 2.39, 0.01)], and “Other disorders of white blood cells” (D72) [< 40 year old age bin (OR 2.40, p = 0.01) versus the 40 + year old age bin (OR 2.14, 0.01)]. Some of these changes in prevalence of diagnoses may be a reflection of the concept of inflammation related aging in Down syndrome.

## Discussion

We identified important differences in the odds of receiving certain diagnoses between our cohort of 1299 individuals with Down syndrome and our control cohort of 2605 individuals without Down syndrome. Previous studies have used analytic methods such as grouping diagnoses of interest for analysis [15]. We employed a novel stepwise approach in our three phase analysis across ICD-10-CM codes at the chapter, subchapter, and diagnostic level in order to avoid selection bias. Using this method, we were able to find the main drivers of protective and risk factors within the disease groupings and organ systems we identified. Our results shed light on the complex interplay between genetics, pathology, and suggests the need for further study into the role of caretakers in the non-emergent health of individuals with Down syndrome.

Individuals with Down syndrome were overall less likely to receive a diagnosis within the chapter of “Certain infectious and parasitic diseases” as previously noted [9]. This protection may be explained by the inclusion in this category of common STIs such as “Herpesviral infections” (OR 0.42, p = 0.01) and “Viral warts” (OR 0.51, p = 0.04). Explanations for this phenomenon in the literature with regards to STIs include potentially delayed onset of sexual maturity in the Down syndrome population, reliance on caregivers to notice signs like warts, and insufficient data on sexual activity in the Down syndrome population [15]. Equal weight in the case of protection against other viral etiologies of infection must be given to the growing understanding of IFNAR influence in the Down syndrome phenotype. Previous work has shown that individuals with Down syndrome may have initial resistance to viral infection due to gene dosage effect of IFNAR1 and IFNAR2, but more serious sequelae to infection once it occurs due to excessive negative regulation of type I IFN, via USP18 [9]. Our analysis additionally shows that individuals were significantly more likely (OR 4.79, p < 0.001) to receive a diagnosis of “Other sepsis,” which includes sepsis due to Staphylococcus aureus, Escherichia coli, and other common bacterial flora. Others have documented large levels of inflammation at baseline in individuals with DS, and perhaps during bacterial infections this may yield a more fertile ground for serious complications like sepsis.

Individuals with Down syndrome were overall less likely to receive a diagnosis within the chapter “Diseases of the skin and subcutaneous tissue.” However, after our stepwise approach to analysis, we found that they had significantly higher odds of receiving a diagnosis of “Alopecia Areata,” “Rosacea,” and “Other follicular disorders” (a category that includes Hidradenitis Suppurutiva) (OR 6.06, p = 0.01, OR 3.11, p < 0.001, OR 3.70, p < 0.001, respectively). However they had reduced odds of a diagnosis of “Androgenic Alopecia,” with none of our cohort of individuals with Down syndrome receiving a diagnosis, and “Acne” (OR 0.66, p = 0.04). This may be a function of the role that family members, home health aides, and other caretakers play in the medical management of individuals with Down syndrome. “Androgenic Alopecia” and “Acne” may be more likely to be discussed during clinical encounters by individuals who are concerned with cosmetic aspects of their health, while “Alopecia Areata” and “Rosacea” may be more concerning to a secondary observer. The pathophysiologies of “Alopecia Areata,” “Rosacea,” and follicular disorders such as Hidradenitis Suppurutiva are less well understood, but are likely to be immune in nature. The association of increased odds of these disorders in our cohort of individuals with Down syndrome, with presumed immune dysregulation as a driving factor, further reinforces these hypotheses.

Individuals with Down syndrome were overall more likely to receive a diagnosis within the chapter “Diseases of the blood and blood forming organs and certain disorders involving the immune mechanism.” However, after our stepwise analysis we found that they had significantly lower odds of receiving a diagnosis of “Iron deficiency anemia” (OR 0.47, p < 0.001). A common presenting symptom of iron deficiency anemia is fatigue. The reduced odds in this case may be due inability to communicate symptoms. Individuals with Down syndrome were more likely to have higher odds of a diagnosis of “Other nutritional anemias, “Purpura and other hemorrhagic conditions,” “Other disorders of white blood cells,” and “Other and unspecified diseases of blood and blood forming organs.” Certain pathologies such as Immune thrombocytopenic purpura (ITP) and Drug rash with eosinophilia and systemic symptoms (DRESS syndrome) are included in the latter two diagnostic categories, which are known to be conditions mediated by a hyperactive immune system. Overall, these findings may be due to more extensive routine laboratory workups for individuals with Down syndrome or previously unexplored downstream effects of the immune dysregulation present in Down syndrome.

In our age binned analysis, younger individuals with Down Syndrome compared to their controls had higher odds of diagnoses of “Other sepsis” (A41), “Rosacea” (L71), “Other follicular disorders” (L73), and “Other disorders of white blood cells” (D72) than older individuals with Down syndrome compared to their own controls. This may be a result of younger individuals with Down syndrome taking more autonomy over their own healthcare, greater clinical attention in recent years to the broad spectrum of pathologies in individuals with Down syndrome, or simply significantly earlier onset of these signs and symptoms compared to age matched groups. Our analysis cannot assess causality but we suggest that this is fruitful ground for further mixed methods analyses of how younger patients with Down syndrome and their caregivers present within the healthcare system as opposed to their older peers.

Limitations of this study included our stepwise approach. We initially set out to investigate how individuals with Down syndrome are diagnosed within the confines of the ICD-10-CM coding system as compared to their peers without Down syndrome. There are over 70,000 codes within the system. By beginning at the chapter level, we may have obscured or glossed over diagnoses with higher prevalence at certain subchapter, sub-subchapter, or diagnostic levels. This top-down approach, as opposed to a more targeted approach, may have uncovered certain incidental findings within the ICD-10-CM coding system that are less relevant on the clinical level. Future studies should consider bracketing analysis of these datasets by decade to account for changes in medical management and a growing understand of the phenotype of the individual with Down syndrome.

In summary, given the data on 1299 individuals with Down syndrome in our health system we posit that dysregulation of the immune system in individuals with Down syndrome has impact on infectious diseases, both lowering the incidence of viral disease, but increasing its severity, likely by molecular mechanism governing regulation of type I IFN response. Akin to this we posit that documented hyperinflammatory state leads to a milieu where sepsis is more likely to occur upon bacterial infections. Our data also suggests inflammation and autoimmune mediated diseases of the skin is exacerbated in individuals with Down syndrome. We also suggest that there is a need for greater clinical attention to non-emergent conditions within the Down syndrome patient population. Finally, we hypothesize that the increased odds of certain diagnoses within a younger Down syndrome cohort may be due to increased inflammation or a combination of other unknown factors. Deeper molecular and biochemical evaluation of the underlying immune regulation will shed deeper light on pathophysiology and allow us to deploy many a drug in the growing arsenal of anti-inflammatories in this population.

## Figures and Tables

**Table 1:** Demographics of Case Cohort vs Control Cohort

	Down Syndrome Cohort	Control Cohort	p-value
**N**	1299	2605	
**Sex (%)**			0.24
Male	604 (46%)	1125 (43%)	
Female	694 (53%)	1480 (57%)	
**Age (%)**			0.93
Mean, Standard Deviation	29, 21	30, 21	
0–1 years	73 (5.62%)	89 (3.00%)	
2–5 years	107 (8.24%)	291 (11%)	
6–10 years	140 (10.78%)	295 (11%)	
11–20 years	202 (15.55%)	327 (13%)	
21–30 years	191 (14.70%)	325 (12%)	
31–40 years	159 (12.24%)	297 (11 %)	
40 + years	427 (32.97%)	981 (38%)	
**Race**			0.89
Non-Hispanic White	403 (50.94%)	905 (51.77%)	
Hispanic	214 (27.05%)	481 (27.52%)	
Black or African American	127 (16.06%)	290 (16.59%)	
Asian	40 (5.06%)	71 (4.06%	
Native Hawaiian	6 (0.76%)	1 (0.06%)	
American Indian	1 (0.12%)	0 (0.00%)	
**Insurance**			0.002
Private Insurance	355 (35.15%)	987 (40.61%)	
Medicare	297 (29.41%)	596 (24.52%)	
Medicaid	351 (34.75%)	840 (34.57%)	
Self-Pay	7 (0.69%)	7 (0.29%)	

**Table 2:** Odds of Receiving a Diagnosis Within ICD-10-CM Chapter Compared with Control

Code Range	Chapter Title	Odds (95% CI)	p-value
V00- Y99	External causes of morbidity	9.28 (7.28–11.00)	< 0.001
I00–99	Diseases of the circulatory system	2.02 (1.76–2.31)	< 0.001
U00- 85	Codes for special purposes	2.00 (1.40–2.88)	< 0.001
G00- 99	Diseases of the nervous system	1.97 (1.71–2.28)	< 0.001
E00- 89	Endocrine, nutritional, metabolic disease	1.89 (1.65–2.16)	< 0.001
H60- 65	Diseases of the ear and mastoid process	1.68 (1.43–1.98)	< 0.001
H00– 59	Diseases of the eye and adnexa	1.52 (1.30–1.78)	< 0.001
F01- 99	Mental, behavioral, and neurodevelopmental disorders	1.47 (1.27–1.70)	< 0.001
R00- 99	Symptoms, signs, and abnormal clinical findings, not elsewhere classified	1.29 (1.11–1.50)	< 0.001
D50- 89	Diseases of the blood and blood forming organs and certain disorders involving the immune mechanism	1.19 (0.98–1.43)	0.08
O00- O9A	Pregnancy, childbirth, and the puerperium	1.12 (0.89–1.41)	0.36
K00- 95	Diseases of the digestive system	1.06 (0.92–1.23)	0.41
P00- 96	Certain conditions originating in the perinatal period	0.99 (0.81–1.21)	0.98
J00- 99	Diseases of the respiratory system	0.97 (0.85–1.11)	0.74
A00- B99	Certain infectious and parasitic diseases	0.87 (0.75–1.00)	0.05
L00- 99	Diseases of the skin and subcutaneous tissue	0.81 (0.70–0.94)	0.01
N00- 99	Diseases of the genitourinary system	0.73 (0.63–0.85)	< 0.001
M00- 99	Diseases of the musculoskeletal system and connective tissue	0.68 (0.58–0.79)	< 0.001
S00- T88	Injury, poisoning and certain other consequences of external causes	0.65 (0.55–0.76)	< 0.001
Z00- 99	Factors influencing health status and contact with health services	0.64 (0.54–0.74)	< 0.001
C00- D49	Neoplasms	0.36 (0.28–0.47)	< 0.001

All statistical analysis was conducted using PyCharm 2023 1.1 Community Edition.

## References

[1] Lagan N, Huggard D, McGrane F, Leahy TR, Franklin O, Roche E, Webb D, O’ Marcaigh A, Cox D, El-Khuffash A, Greally P, Balfe J, Molloy EJ. Multiorgan involvement and management in children with Down syndrome. Acta Paediatr. 2020;109(6):1096–111. 10.1111/apa.15153. 31899550

[2] Bull MJ, Down Syndrome. N Engl J Med. 2020;382(24):2344–52. 10.1056/NEJMra1706537. 32521135

[3] Malle L, Bogunovic D. Down syndrome and type I interferon: not so simple. Curr Opin Immunol. 2021;72:196–205. 10.1016/j.coi.2021.06.006. 34174697 PMC8578175

[4] Antonarakis SE, Skotko BG, Rafii MS, Strydom A, Pape SE, Bianchi DW, Sherman SL, Reeves RH. Down syndrome. Nat Rev Dis Primers. 2020;6(1):9. 10.1038/s41572-019-0143-7. 32029743 PMC8428796

[5] Arumugam A, Raja K, Venugopalan M, Chandrasekaran B, Kovanur Sampath K, Muthusamy H, Shanmugam N. Down syndrome-A narrative review with a focus on anatomical features. Clin Anat. 2016;29(5):568–77. 10.1002/ca.22672. 26599319

[6] Lagan N, Huggard D, Mc Grane F, Leahy TR, Franklin O, Roche E, Webb D, O’ Marcaigh A, Cox D, El-Khuffash A, Greally P, Balfe J, Molloy EJ. Multiorgan involvement and management in children with Down syndrome. Acta Paediatr. 2020;109(6):1096–111. 10.1111/apa.15153. 31899550

[7] Roizen NJ, Patterson D. Down’s syndrome. Lancet. 2003;361(9365):1281–9. 10.1016/S0140-6736(03)12987-X. 12699967

[8] Araya P, Waugh KA, Sullivan KD, Núñez NG, Roselli E, Smith KP, Granrath RE, Rachubinski AL, Enriquez Estrada B, Butcher ET, Minter R, Tuttle KD, Bruno TC, Maccioni M, Espinosa JM. Trisomy 21 dysregulates T cell lineages toward an autoimmunity-prone state associated with interferon hyperactivity. Proc Natl Acad Sci U S A. 2019;116(48):24231–41. 10.1073/pnas.1908129116. 31699819 PMC6883781

[9] Malle L, Martin-Fernandez M, Buta S, Richardson A, Bush D, Bogunovic D. Excessive negative regulation of type I interferon disrupts viral control in individuals with Down syndrome. Immunity. 2022;55(11):2074–2084e5. 10.1016/j.immuni.2022.09.007. 36243008 PMC9649881

[10] Kong XF, Worley L, Rinchai D, Bondet V, Jithesh PV, Goulet M, Nonnotte E, Rebillat AS, Conte M, Mircher C, Gürtler N, Liu L, Migaud M, Elanbari M, Habib T, Ma CS, Bustamante J, Abel L, Ravel A, Lyonnet S, Munnich A, Duffy D, Chaussabel D, Casanova JL, Tangye SG, Boisson-Dupuis S, Puel A. Three Copies of Four Interferon Receptor Genes Underlie a Mild Type I Interferonopathy in Down Syndrome. J Clin Immunol. 2020;40(6):807–19. 10.1007/s10875-020-00803-9. 32572726 PMC7418179

[11] Sullivan KD, Lewis HC, Hill AA, Pandey A, Jackson LP, Cabral JM, Smith KP, Liggett LA, Gomez EB, Galbraith MD, DeGregori J, Espinosa JM. Trisomy 21 consistently activates the interferon response. Elife. 2016;5:e16220. 10.7554/eLife.16220. 27472900 PMC5012864

[12] Malle L, Patel RS, Martin-Fernandez M, Stewart OJ, Philippot Q, Buta S, Richardson A, Barcessat V, Taft J, Bastard P, Samuels J, Mircher C, Rebillat AS, Maillebouis L, Vilaire-Meunier M, Tuballes K, Rosenberg BR, Trachtman R, Casanova JL, Notarangelo LD, Gnjatic S, Bush D, Bogunovic D. Autoimmunity in Down’s syndrome via cytokines, CD4 T cells and CD11c+ B cells. Nature. 2023;615(7951):305–14. 10.1038/s41586-023-05736-y. 36813963 PMC9945839

[13] About ATLAS. | Mount Sinai Data Warehouse. Labs.icahn.mssm.edu 2021 [cited 2023 Nov 16]. Available from: https://labs.icahn.mssm.edu/msdw/about-atlas/.

[14] ICD-10-CM. Cdc.goc. 2020. Avaialble from: https://icd10cmtool.cdc.gov/I.

[15] Chicoine B, Rivelli A, Fitzpatrick V, Chicoine L, Jia G, Rzhetsky A. Prevalence of Common Disease Conditions in a Large Cohort of Individuals With Down Syndrome in the United States. J Patient Cent Res Rev. 2021;8(2):86–97. 10.17294/2330-0698.1824. 33898640 PMC8060042

[16] Malle L, Gao C, Hur C, Truong HQ, Bouvier NM, Percha B, Kong XF, Bogunovic D. Individuals with Down syndrome hospitalized with COVID-19 have more severe disease. Genet Med. 2021;23(3):576–80. 10.1038/s41436-020-01004-w. 33060835 PMC7936948

[17] Verstegen RHJ, Chang KJJ, Kusters MAA. Clinical implications of immune-mediated diseases in children with Down syndrome. Pediatr Allergy Immunol. 2020;31(2):117–23. 10.1111/pai.13133. 31599041

[18] Zhang Y, Che M, Yuan J, Yu Y, Cao C, Qin XY, Cheng Y. Aberrations in circulating inflammatory cytokine levels in patients with Down syndrome: a meta-analysis. Oncotarget. 2017;8(48):84489–96. 10.18632/oncotarget.21060. 29137441 PMC5663613

